# Diagnostic ability of macular microvasculature with swept-source OCT angiography for highly myopic glaucoma using deep learning

**DOI:** 10.1038/s41598-023-32164-9

**Published:** 2023-03-30

**Authors:** Yun Jeong Lee, Sukkyu Sun, Young Kook Kim, Jin Wook Jeoung, Ki Ho Park

**Affiliations:** 1 Department of Ophthalmology, Seoul National University Hospital, Seoul National University College of Medicine, Seoul, Korea; 2 Biomedical Research Institute, Seoul National University Hospital, Seoul, Korea

**Keywords:** Medical imaging, Diagnostic markers, Optic nerve diseases

## Abstract

Macular OCT angiography (OCTA) measurements have been reported to be useful for glaucoma diagnostics. However, research on highly myopic glaucoma is lacking, and the diagnostic value of macular OCTA measurements versus OCT parameters remains inconclusive. We aimed to evaluate the diagnostic ability of the macular microvasculature assessed with OCTA for highly myopic glaucoma and to compare it with that of macular thickness parameters, using deep learning (DL). A DL model was trained, validated and tested using 260 pairs of macular OCTA and OCT images from 260 eyes (203 eyes with highly myopic glaucoma, 57 eyes with healthy high myopia). The DL model achieved an AUC of 0.946 with the OCTA superficial capillary plexus (SCP) images, which was comparable to that with the OCT GCL+ (ganglion cell layer + inner plexiform layer; AUC, 0.982; *P* = 0.268) or OCT GCL++ (retinal nerve fiber layer + ganglion cell layer + inner plexiform layer) images (AUC, 0.997; *P* = 0.101), and significantly superior to that with the OCTA deep capillary plexus images (AUC, 0.779; *P* = 0.028). The DL model with macular OCTA SCP images demonstrated excellent and comparable diagnostic ability to that with macular OCT images in highly myopic glaucoma, which suggests macular OCTA microvasculature could serve as a potential biomarker for glaucoma diagnosis in high myopia.

## Introduction

Glaucoma, a leading cause of irreversible blindness^1^, is a significant global disease burden that in 2020 was estimated to affect 76 million people^2^. Making matters worse, the global prevalence of myopia, which is a risk factor for glaucoma^3^, is expected to increase to about 5 billion people by 2050^4^, which in turn could lead to increased visual impairment resulting from myopia-associated diseases including glaucoma and others. Certainly, the growing importance of accurate diagnosis and proper management of glaucoma in myopic populations cannot be overstated.

The diagnosis of glaucoma in myopic individuals, especially those with high myopia, however, is often challenging due to myopia-induced structural changes. Peripapillary retinal nerve fiber layer (RNFL) thickness, as measured with optical coherence tomography (OCT), is thinner in myopic eyes than in non-myopic eyes^5^, and optic nerve head (ONH) deformations such as optic disc tilt or peripapillary atrophy could hinder its accurate evaluation^6^. Furthermore, axial elongation causes temporalization of RNFL distribution, resulting in abnormal peripapillary RNFL profiles^5,7^. In an effort to overcome such limitations, macular measurements of ganglion cell-inner plexiform layer (GCIPL) and ganglion cell complex (GCC; RNFL + ganglion cell layer [GCL] + inner plexiform layer [IPL]) have been suggested as alternative parameters, which have shown comparable or even better diagnostic ability than that of peripapillary RNFL thickness for glaucoma in myopic or highly myopic eyes^8–12^.

Recently, OCT angiography (OCTA) imaging has demonstrated its usefulness to glaucoma diagnostics by revealing peripapillary and macular vessel density decrement and choroidal microvasculature dropout^13–15^. Along with the advancement of imaging technology, recent application of deep learning (DL) within the field of ophthalmology, including the glaucoma subfield, has shown potential utility for disease screening, diagnosis and prognosis prediction^16–19^. A Vision Transformer (ViT), which is an extended, recent application of Transformer to computer vision, has attained excellent results in image classification and has even outperformed convolutional neural network when pre-trained^20^. Moreover, it has shown its utility in ophthalmology as well^21,22^.

Notwithstanding the ongoing efforts to apply macular OCT measurements for improved diagnostic accuracy in cases of highly myopic glaucoma^8,9,11,12^, diagnosis remains challenging in clinical practice, which necessitates a novel, discriminative diagnostic parameter to overcome the challenges. They include false-positive results, which could be caused by macular thinning in myopic eyes arising from mechanical stretching of the globe along with axial length (AXL) elongation. In addition, myopic maculopathy (e.g., myopic foveoschisis) could influence macular OCT measurement. Moreover, myopic eyes, especially highly myopic eyes, are susceptible to segmentation error and low signal strength in OCT. Although there were several studies that revealed the usefulness of macular OCTA measurements for the diagnosis of glaucoma^23–27^, research on highly myopic glaucoma is lacking, and the diagnostic value of macular OCTA measurements versus OCT parameters remains inconclusive^24,26–39^. The aim of our study, therefore, was to evaluate the diagnostic ability of the macular microvasculature assessed with swept-source OCTA (SS-OCTA) for highly myopic glaucoma and to compare it with that of macular thickness parameters assessed with SS-OCT, using DL.

## Results

The demographics and clinical characteristics of the eyes with highly myopic glaucoma and the healthy highly myopic eyes are compared in Table 1. There were no significant inter-group differences with regard to age, gender, intraocular pressure (IOP), spherical equivalent (SE), AXL or central corneal thickness (CCT), the one exception being visual field (VF) mean deviation, in which the highly myopic glaucomatous eyes were significantly worse than the healthy highly myopic eyes (− 5.38 ± 5.49 dB vs. − 0.02 ± 1.67 dB, respectively, *P* < 0.001). Of the 260 pairs of OCTA and OCT images from the 260 eyes, including 203 eyes with highly myopic glaucoma and 57 healthy highly myopic eyes, the number of images used for training, validation, and test datasets were as follows: 127 images from the highly myopic glaucomatous eyes and 38 from the healthy highly myopic eyes for the training dataset; 37 from the highly myopic glaucomatous eyes and 9 from the healthy highly myopic eyes for the validation dataset; 39 from the highly myopic glaucomatous eyes and 10 from the healthy highly myopic eyes for the test dataset.

**Table 1:** Comparison of demographics and clinical characteristics between highly myopic glaucomatous eyes and healthy highly myopic eyes.

	Highly myopic glaucoma (n = 203)	Healthy high myopia (n = 57)	*P* value
Age (years)	46.6 ± 9.8	42.7 ± 14.1	0.052*
Female, no. (%)	81 (39.9)	28 (49.1)	0.213^**†**^
Intraocular pressure (mmHg)	13.8 ± 2.1	14.1 ± 2.8	0.431*
Spherical equivalent (D)	− 8.1 ± 1.3	− 8.5 ± 1.6	0.177*
Axial length (mm)	26.9 ± 0.7	26.8 ± 0.6	0.254*
Central corneal thickness (µm)	546.5 ± 40.1	551.8 ± 33.5	0.413*
Visual field mean deviation (dB)	− 5.38 ± 5.49	− 0.02 ± 1.67	< 0.001*

Data are mean ± standard deviation unless otherwise indicated.

Boldface indicates *P* < 0.05.

*Student’s *t*-test.

^†^Chi-square test.

**Figure 1:**
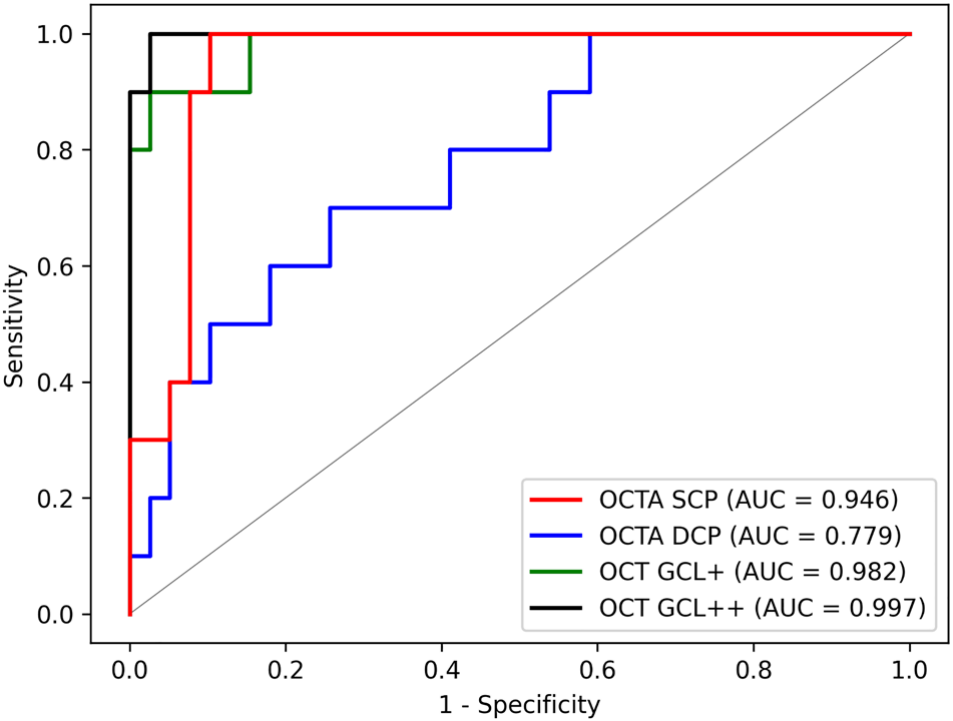
Receiver operating characteristic (ROC) curves of deep learning (DL) models for distinguishing highly myopic glaucoma from healthy high myopia. AUC, area under the receiver operating characteristic curve; DCP, deep capillary plexus; GCL+, ganglion cell layer + inner plexiform layer; GCL++, retinal nerve fiber layer + ganglion cell layer + inner plexiform layer; SCP, superficial capillary plexus.

The performance of the DL model in distinguishing highly myopic glaucoma from healthy high myopia is shown in Table 2. For the OCTA superficial capillary plexus (SCP) images, our DL model achieved an area under the receiver operating characteristic (ROC) curve (AUC) of 0.946 (95% confidence interval [CI] 0.885–1.000), an accuracy of 0.837 (95% CI 0.703–0.927), a sensitivity of 0.923 (95% CI 0.795–1.000), and a specificity of 1.000 (95% CI 1.000–1.000). Using the OCTA deep capillary plexus (DCP) images, an AUC of 0.779 (95% CI 0.623–0.936), an accuracy of 0.816 (95% CI 0.680–0.912), a sensitivity of 0.769 (95% CI 0.333–0.974), and a specificity of 0.800 (95% CI 0.500–1.000) were obtained. Regarding the OCT images, an AUC of 0.982 (95% CI 0.949–1.000), an accuracy of 0.939 (95% CI 0.831–0.987), a sensitivity of 0.974 (95% CI 0.795–1.000), and a specificity of 1.000 (95% CI 0.800–1.000) were obtained with the OCT GCL+ images, and an AUC of 0.997 (95% CI 0.990–1.000), an accuracy of 0.980 (95% CI 0.892–1.000), a sensitivity of 1.000 (95% CI 0.923–1.000), and a specificity of 1.000 (95% CI 1.000–1.000) with the OCT GCL++ images. Comparing the DL model’s performances using DeLong’s test, the AUC with the OCTA SCP images was comparable to that with the OCT GCL+ (0.946 with OCTA SCP vs. 0.982 with OCT GCL+, *P* = 0.268) or OCT GCL++ images (0.946 with OCTA SCP vs. 0.997 with OCT GCL++, *P* = 0.101), and was significantly superior to that with the OCTA DCP images (0.946 with OCTA SCP vs. 0.779 with OCTA DCP, *P* = 0.028). Further comparison of the OCTA DCP and OCT parameters’ AUCs revealed that with OCTA DCP also was significantly worse than that with OCT GCL+ (0.779 with OCTA DCP vs. 0.982 with OCT GCL+, *P* = 0.014) or OCT GCL++ (0.779 with OCTA DCP vs. 0.997 with OCT GCL++, *P* = 0.006). The AUCs with the two OCT parameters, however, showed no significant difference (0.982 with OCT GCL+ vs. 0.997 with OCT GCL++, *P* = 0.290). The ROC curves for each of the images are shown in Fig. 1.

**Table 2:** Performance of deep learning model in distinguishing highly myopic glaucoma from healthy high myopia.

	AUC (95% CI)	Accuracy (95% CI)	Sensitivity (95% CI)	Specificity (95% CI)	*P* value*		
					OCTA DCP	OCT GCL+	OCT GCL++
OCTA SCP	0.946 (0.885–1.000)	0.837 (0.703–0.927)	0.923 (0.795–1.000)	1.000 (1.000–1.000)	**0.028**	0.268	0.101
OCTA DCP	0.779 (0.623–0.936)	0.816 (0.680–0.912)	0.769 (0.333–0.974)	0.800 (0.500–1.000)	–	**0.014**	**0.006**
OCT GCL+	0.982 (0.949–1.000)	0.939 (0.831–0.987)	0.974 (0.795–1.000)	1.000 (0.800–1.000)	–	–	0.290
OCT GCL++	0.997 (0.990–1.000)	0.980 (0.892–1.000)	1.000 (0.923–1.000)	1.000 (1.000–1.000)	–	–	–

*AUC* area under the receiver operating characteristic curve, *CI* confidence interval, *DCP* deep capillary plexus, *GCL*+ ganglion cell layer + inner plexiform layer, *GCL*++ retinal nerve fiber layer + ganglion cell layer + inner plexiform layer, *OCT* optical coherence tomography, *OCTA* optical coherence tomography angiography, *SCP* superficial capillary plexus.

Boldface indicates *P* < 0.05.

*DeLong’s test.

**Figure 2:**
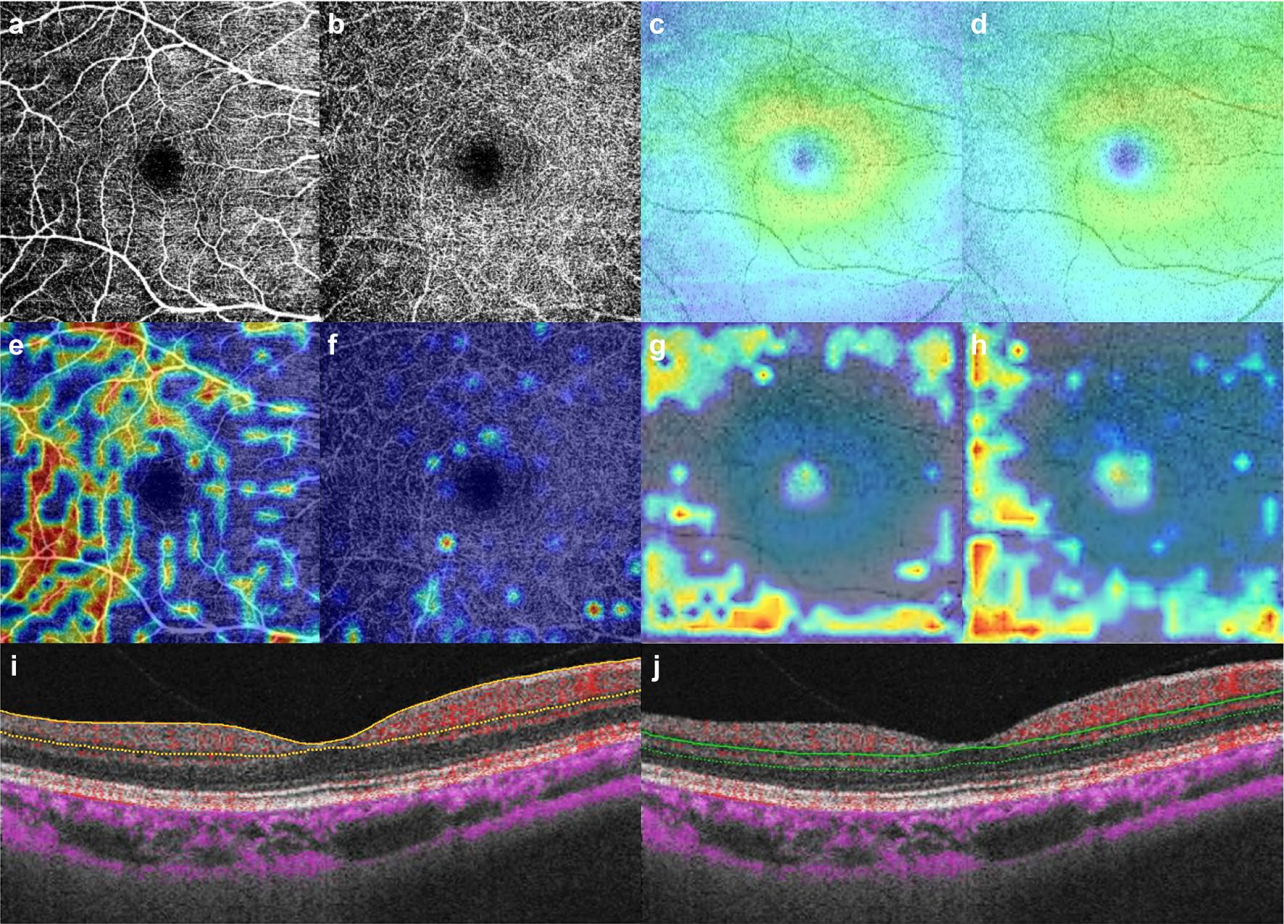
Macular OCT angiography (OCTA) and OCT images with heatmaps for prediction of highly myopic glaucoma by trained deep learning (DL) model. (**a**) OCTA superficial capillary plexus (SCP) image. (**b**) OCTA deep capillary plexus (DCP) image. (**c**) OCT GCL+ (ganglion cell layer + inner plexiform layer) image. (**d**) OCT GCL++ (retinal nerve fiber layer + ganglion cell layer + inner plexiform layer) image. (**e**–**h**) Heatmaps for OCTA SCP, OCTA DCP, OCT GCL+ and OCT GCL++ images, respectively (shown in first row). The heatmap revealed that the areas of microvasculature dropout with decreased vessel density were mainly highlighted in the OCTA SCP image (**e**), those areas being the main contributor to the model’s determinations. However, the heatmap was poorly activated in the OCTA DCP image (**f**). In the heatmaps for the OCT GCL+ (**g**) and GCL++ images (**h**), the regions where their thickness was decreased were highlighted. Corresponding B-scans with flow overlay (in red) of SCP (between the yellow lines; from 2.6 μm below the internal limiting membrane to 15.6 μm below the junction between the inner plexiform and the inner nuclear layers) (**i**) and DCP (between the green lines; from 15.6 μm below the inner plexiform and inner nuclear layers to 70.2 μm below them) (**j**).

In the final stage, heatmaps, as presented with an example in Fig. 2, were generated with gradient-weighted class activation mapping (Grad-CAM) to visualize the regions of the images that had contributed to the trained DL model’s decisions. The heatmaps demonstrated that the areas of microvasculature dropout with decreased vessel density were mainly highlighted in the OCTA SCP images. Also, in the OCT GCL+ and GCL++ images, the regions where their thickness was decreased were highlighted. For the OCTA DCP images, however, the heatmaps were poorly activated.

For external validation, a total of 108 pairs of macular OCTA and OCT images from 108 eyes (92 eyes with highly myopic glaucoma and 16 eyes with healthy high myopia) were used to differentiate highly myopic glaucoma from healthy high myopia. The demographics and clinical characteristics of the eyes of the two groups showed no significant differences except for VF mean deviation, in which highly myopic glaucomatous eyes were significantly worse than healthy highly myopic eyes (− 5.07 ± 4.91 dB vs. − 0.30 ± 1.18 dB, respectively, *P* < 0.001; Supplementary Table S1). The DL model’s performance statistics in distinguishing highly myopic glaucoma from healthy high myopia with the external datasets are provided in Supplementary Table S2. For the OCTA SCP images, the DL model achieved an AUC of 0.873 (95% CI 0.807–0.940), an accuracy of 0.807 (95% CI 0.724–0.873), a sensitivity of 0.462 (95% CI 0.154–0.539), and a specificity of 0.903 (95% CI 0.365–0.903). Using the OCTA DCP images, an AUC of 0.794 (95% CI 0.687–0.902), an accuracy of 0.815 (95% CI 0.734–0.880), a sensitivity of 0.308 (95% CI 0.154–0.615), and a specificity of 0.957 (95% CI 0.591–0.957) were obtained. As for the OCT images, an AUC of 0.970 (95% CI 0.941–1.000), an accuracy of 0.935 (95% CI 0.871–0.974), a sensitivity of 0.923 (95% CI 0.423–1.000), and a specificity of 0.939 (95% CI 0.854–0.988) were obtained with the OCT GCL+ images, and an AUC of 0.843 (95% CI 0.722–0.964), an accuracy of 0.817 (95% CI 0.696–0.905), a sensitivity of 0.353 (95% CI 0.294–0.824), and a specificity of 1.000 (95% CI 0.163–1.000) with the OCT GCL++ images.

## Discussion

Evaluating the diagnostic ability of the macular microvasculature assessed with SS-OCTA for highly myopic glaucoma and comparing it with that of macular thickness parameters measured with SS-OCT using DL, we found that our DL model with macular SCP images demonstrated excellent and comparable performance to that with macular thickness parameters for differentiating highly myopic glaucoma from healthy high myopia.

Our results are in line with previous studies that reported promising glaucoma-diagnostic performance of macular OCTA SCP^24,27,34,36,37^ and limited ability of macular OCTA DCP^24,34^, both of which conclusions are supported by Takusagawa et al.’s^40^ report that the superficial vascular complex is the preferential level at which perfusion decreases in glaucoma. These findings were reconfirmed in the present study’s heatmaps, on which the areas of microvasculature dropout were mainly highlighted in the OCTA SCP images, but which were poorly activated for the OCTA DCP images. Although Lee et al.’s study^38^, which to our knowledge is the only one to have evaluated the glaucoma-diagnostic ability of OCTA for highly myopic eyes, concluded that average SCP vessel density has a low diagnostic power, they had included only a small number of patients, which fact makes generalization of the results to other populations problematic. Our study’s further comparison of the diagnostic ability of macular OCTA and OCT images for highly myopic glaucoma revealed that the performance with macular OCTA SCP images was comparable to that with macular OCT images, which finding is consistent with earlier reports demonstrating a comparable glaucoma-diagnostic ability of macular OCTA SCP to that of OCT GCC^27,36,37^. As opposed to our results, however, several studies have reported lower diagnostic ability of macular OCTA measurements as compared with OCT GCIPL or GCC for glaucoma^26,28–35,39^. This discrepancy might have arisen from differences between the respective studies’ OCTA devices, which use different algorithms and have different imaging performances^41^. Another possible factor could have been the different sizes of macular area analyzed; indeed, Penteado et al.’s study suggested that the diagnostic accuracy of vessel density for differentiating between healthy eyes and those with mild glaucoma differs according to the macular OCTA scan size^36^. Also, some of the relevant earlier studies did not make statistical comparisons of diagnostic power between parameters, which fact renders comparison of their true performances difficult. In our further attempt to combine both layers of OCTA SCP and DCP by arraying the 4 images of OCTA SCP, OCTA DCP, OCT GCL+ and OCT GCL++ 4 × 4 as a single, combined image, the DL model achieved an AUC of 0.974, which was inferior to that with either OCT GCL+ or OCT GCL++ images alone. Therefore, we investigated the DL model’s performance using the respective images, rather than combining those of SCP and DCP.

Assessment of macular OCT measurements of GCIPL and GCC is useful in myopic glaucoma diagnostics, since the macular region is thought to be less distorted in myopia, whereas the ONH, due to deformation, is difficult to evaluate. Macular OCT measurements, however, are not without their limitations. Macular thinning in myopic eyes^42–44^, which arises from mechanical stretching of the globe along with AXL elongation, could result in false-positive results in macular OCT^45^. Also, myopic eyes, especially highly myopic eyes, are prone to segmentation error and low signal strength in OCT^46,47^. Moreover, myopic maculopathy such as myopic foveoschisis could affect macular OCT measurements^48^. The macular OCTA microvasculature, which might be less vulnerable to those measurement problems associated with high myopia than is OCT, could be an alternative solution in circumstances where diagnoses of glaucoma by conventional imaging such as OCT are uncertain. Furthermore, combining the macular OCTA microvasculature’s advantage for highly myopic glaucoma with that for advanced glaucoma over OCT parameters (e.g., macular OCTA vessel density lacks the measurement floor effect encountered with OCT measurements^49^) could make the macular OCTA microvasculature a promising tool for monitoring of disease progression in cases of advanced glaucoma with high myopia.

The strength of our study is that, to our best knowledge, it is the first to evaluate the diagnostic value of the macular microvasculature for highly myopic glaucoma in a large population. Also, whereas most of the earlier OCTA studies on glaucoma focused on the SCP only, we also analyzed the DCP to determine its comparative diagnostic ability. Other main strengths are our utilization of a DL method enabling automated and fast analysis of a large amount of imaging data and our adoption of a state-of-the-art ViT model as a DL model. Moreover, we took advantages of SS-OCT imaging, which are faster scanning speed with larger imaging areas, reduced motion artifacts, and incorporation of longer wavelength resulting in reduced signal attenuation and increased depth of resolution compared with conventional spectral-domain OCT^50^.

The limitations of the present study include its retrospective design and lack of comparisons of DL model performance among groups of differing disease severity or different OCTA image sizes with various OCTA devices. Further studies of prospective design that additionally explore these aspects would expand our knowledge of the diagnostic value of OCTA for highly myopic glaucoma; as for the types of OCTA devices, AngioVue RTVue XR Avanti (Optovue), Spectralis OCT Angiography (Heidelberg Engineering), PLEX Elite 9000 (Carl Zeiss Meditech) as well as the present study’s deep range imaging (DRI) OCT Triton (Topcon), which utilize different algorithms and settings (Supplementary Table S3), have been used in the previous research^24,26,27,29–33,35,51^. Another limitation is that the different OCT and OCTA image sizes could have affected the evaluation due to the different information they might contain. Given that we had retrospectively collected the data, we were unable to control for the scan size. Future studies utilizing OCT and OCTA images of the same size are required. In addition, further research with larger datasets is necessary, since the small size of the present datasets resulted in large CIs in our study. Also, it was impracticable to obtain better-quality images, because making their resolution better than that of the original ones was technically not possible with preprocessing. Notwithstanding this limitation regarding image quality, which is innate to OCTA devices, we had included only images of good quality by excluding others based on the following criteria: motion or blink artifacts, defocus, segmentation error, or quality score less than 50 (as indicated in the “Methods” section). Development of imaging devices that provide better imaging quality is required for more accurate disease evaluation. Longitudinal studies to determine the association between macular microvasculature change and glaucoma progression in high myopia would also provide another useful means of evaluating the progression of highly myopic glaucoma. Regarding our external validation, the DL model showed similar or slightly lesser performance with the external datasets than with the internal ones, except for the sensitivity values, which showed pronounced decrements with the external ones (sensitivity of 0.462 vs. 0.923 with the OCTA SCP images, 0.308 vs. 0.769 with the OCTA DCP images, 0.923 vs. 0.974 with the OCT GCL+ images, and 0.353 vs. 1.000 with the OCT GCL++ images). The lesser performance of the DL model with the external datasets suggests that it had been biased to the training dataset, which might have been due to the change in the distribution of the data, a domain shift, which could have negatively affected the model’s performance^52^. Indeed, further comparison of the internal and external datasets revealed that they differed in the distribution of demographics and clinical characteristics such as age, gender, SE, AXL, VF mean deviation, and glaucomatous eyes, as demonstrated with distribution plots in Supplementary Figure S1. Especially, among the characteristics, mean age showed significant differences between the internal and external datasets for highly myopic glaucomatous eyes (*P* = 0.001) (Supplementary Table S4). Domain shift is a typical phenomenon that occurs when DL models are evaluated with datasets dissimilar from the training data distribution, which can be caused, for example, by changes in disease prevalence or testing procedures^52^. In fact, Yu et al.’s systematic review reported that the vast majority of published external validation studies of DL algorithms for image-based radiologic diagnostics demonstrated diminished algorithm performance on the external dataset, with some reporting a substantial performance decrease^53^. Earlier studies using retinal fundus photographs, for instance, also have reported worse performance of the DL model with external validation^54,55^. Future studies are required to further evaluate its external validity.

Notwithstanding the limitations, our study is of great importance in that it newly investigated and verified the diagnostic value of the macular OCTA microvasculature for highly myopic glaucoma, which had been lacking in previous studies. Furthermore, our results are valuable in that, by revealing the DL model’s excellent and comparable diagnostic ability with macular OCTA SCP images to that with macular OCT images in highly myopic glaucoma, we showed the potential of the macular OCTA microvasculature as a novel, alternative biomarker for glaucoma diagnosis in high myopia. This is especially meaningful, because with conventional diagnostic methods including OCT, the diagnosis of highly myopic glaucoma is still often challenging in clinical practice. We believe that the findings of our study should benefit clinicians in helping them to make accurate diagnoses, and treatment decisions thereby, for eyes with highly myopic glaucoma.

In conclusion, the DL model with macular OCTA SCP images demonstrated excellent and comparable diagnostic ability to that with macular OCT images for highly myopic glaucoma, which suggests that the macular OCTA microvasculature could serve as a potential alternative biomarker for glaucoma diagnosis in cases of high myopia.

## Methods

This retrospective, cross-sectional study enrolled patients who had visited Seoul National University Hospital (SNUH) in South Korea between July 2017 and September 2021. The study was approved by the Institutional Review Board of SNUH (IRB no. 2111-164-1276), and the study protocol followed the tenets of the Declaration of Helsinki. The requirement for informed consent from the study subjects was waived by the IRB of SNUH due to the retrospective nature of the study.

### Participants

All of the participants underwent a complete ophthalmologic examination, including measurements of best-corrected visual acuity (BCVA), refraction, CCT (Pocket II Pachymeter Echograph; Quantel Medical, Inc., Clermont-Ferrand, France) and AXL (Axis II PR; Quantel Medical, Inc., Bozeman, MT, USA), along with Goldmann applanation tonometry, slit-lamp biomicroscopy, gonioscopy, dilated fundus examination, stereo disc photography, red-free RNFL photography (Visucam; Carl Zeiss Meditec, Inc., Dublin, CA, USA), SS-OCTA and SS-OCT (DRI OCT Triton; Topcon, Tokyo, Japan), and standard automated perimetry (Humphrey Field Analyzer II; 24–2 Swedish Interactive Threshold Algorithm; Carl Zeiss Meditec).

For inclusion in the study, subjects were required to meet the following criteria: BCVA of 20/40 or better, normal anterior chamber and open angle on slit-lamp and gonioscopic examinations, high myopia with SE of less than − 6.0 diopters (D) or AXL of more than 26.0 mm, and astigmatism within ± 3D. Glaucoma was defined as the presence of glaucomatous optic disc changes (i.e., neuroretinal rim notching, thinning) and RNFL defects on red-free RNFL photography with corresponding glaucomatous VF defect. RNFL defects on red-free RNFL photography were defined as diverging, arched, or wedge-shaped, and wider than the major retinal vessel at a distance of 1-disc diameter from the edge of the disc^56,57^. Glaucomatous VF defect was defined as (1) glaucoma hemifield test values outside the normal limits; (2) 3 or more abnormal points with a probability of being normal of *P* < 5%, of which at least 1 point has a pattern deviation of *P* < 1%; or (3) a pattern standard deviation of *P* < 5%, as confirmed by 2 consecutive reliable tests (fixation loss rates ≤ 20%, false-positive and false-negative error rates ≤ 25%). The criteria for healthy eyes were IOP of 21 mmHg or less, no history of elevated IOP, optic disc of normal appearance, no RNFL defect on red-free RNFL photography, and normal VF results. Color disc and red-free RNFL photographs were evaluated by 2 glaucoma specialists (Y.J.L. and K.H.P.) masked to all clinical information.

The exclusion criteria were defined, in reference to the criteria from earlier studies^58,59^, as SE of less than − 12.0D (in order to exclude extremely highly myopic eyes prone to segmentation error and low signal strength in OCT as well as myopic maculopathy which could affect macular OCT measurements); history of ocular surgery other than uncomplicated cataract surgery; ocular inflammation or trauma; ocular conditions other than glaucoma possibly affecting macular vessel density or macular thickness, and retinal disease (e.g., diabetic retinopathy, retinal vein occlusion, age-related macular degeneration) or neurologic disease (e.g., pituitary tumor) possibly causing VF defect.

### OCT angiography and OCT imaging

All of the participants underwent OCTA and OCT imaging by SS-OCT (DRI OCT Triton; Topcon, Tokyo, Japan) with a central wavelength of 1050 nm and an acquisition speed of 100,000 A-scans per second. The OCTA scans were obtained from 4.5 × 4.5 mm cubes centered on the fovea. En-face images of the vascular structure in the SCP and DCP were automatically generated by the built-in analysis software (IMAGEnet 6, v1.25.16650); the SCP was delineated from 2.6 μm below the internal limiting membrane to 15.6 μm below the junction between the inner plexiform and the inner nuclear layers; the DCP was delineated from 15.6 μm below the inner plexiform and inner nuclear layers to 70.2 μm below them. The corresponding OCT B-scan images of SCP and DCP are provided in Fig. 2. For the OCT imaging, a wide-field scan protocol (12 × 9 mm^2^, 512 × 256 pixels) was applied, by which the GCL+ (GCL + IPL) and GCL++ (RNFL + GCL + IPL) layer thicknesses were measured over the central 6 × 6 mm^2^ macular region with the built-in software. OCTA or OCT images with motion or blink artifacts, defocus, segmentation error, or of quality score (provided automatically by the OCT device) less than 50, in reference to the criteria from previous studies^56,60^, were excluded. As for detection of glaucomatous structural change, defects on the DRI OCT wide-field thickness map were defined as an arcuate or wedge-shaped diverging dark-blue (for RNFL and GCL+) or light-blue (for GCL++) area surrounding an abrupt color-scale change, with minimum defect size being larger than the diameter of a major retinal vessel^57^. All of the images were independently assessed by 2 investigators (Y.J.L and Y.K.K) for diagnosis and final eligibility, and discrepancies were resolved through discussion and consensus or, if needed, adjudication by a third investigator (K.H.P).

### Image dataset and preprocessing

Among the 357 pairs of eligible OCTA and OCT images obtained from IMAGEnet 6, a total of 260 pairs of the images from 260 eyes (203 eyes with highly myopic glaucoma and 57 eyes with healthy high myopia) were included in the subsequent analyses. A total of 97 eyes were excluded (67 eyes with highly myopic glaucoma due to poor-quality images [n = 40], segmentation error [n = 26] or epiretinal membrane [n = 1], and 30 eyes with healthy high myopia due to poor-quality images [n = 16], segmentation error [n = 13] or epiretinal membrane [n = 1]).

To obtain images of the central 6 × 6 mm^2^ macular region, which is automatically demarcated from wide-field scan OCT images by the IMAGEnet 6 software’s dotted square, we conducted image preprocessing using thresholding, erosion, dilation, inversion, sliding, and cropping techniques sequentially. First, the images were converted to grayscale ones. Second, thresholding was applied with a threshold pixel-intensity value of 70 to convert the grayscale images into binary (i.e., black and white) images. Third, dotted squares were converted to solid squares with erosion and dilation using the 3 × 3 kernel. Fourth, inversion was performed. Fifth, sliding of a 100 × 100 pixel-square was performed to find the coordinates where maximum overlap occurs between the two squares. Sixth and finally, the images of the overlapping area were obtained by cropping. Subsequently, each of the OCTA and OCT images was resized to 384 × 384 pixels using bicubic interpolation.

### Deep learning model development

The entire dataset was randomly split into training, validation, and test datasets. The training dataset was used to train the DL model, the validation dataset to determine when to end the training, and the test dataset to evaluate the model’s performance. We utilized a ViT model that recently had been applied for image classification and achieved state-of-the-art, top-1 accuracy among models classified with ImageNet^20^. Of the several ViT models, we used a ViT-Base model (ViT-B/16) that was pre-trained with ImageNet-21k and fine-tuned with ImageNet-1k.

**Figure 3:**
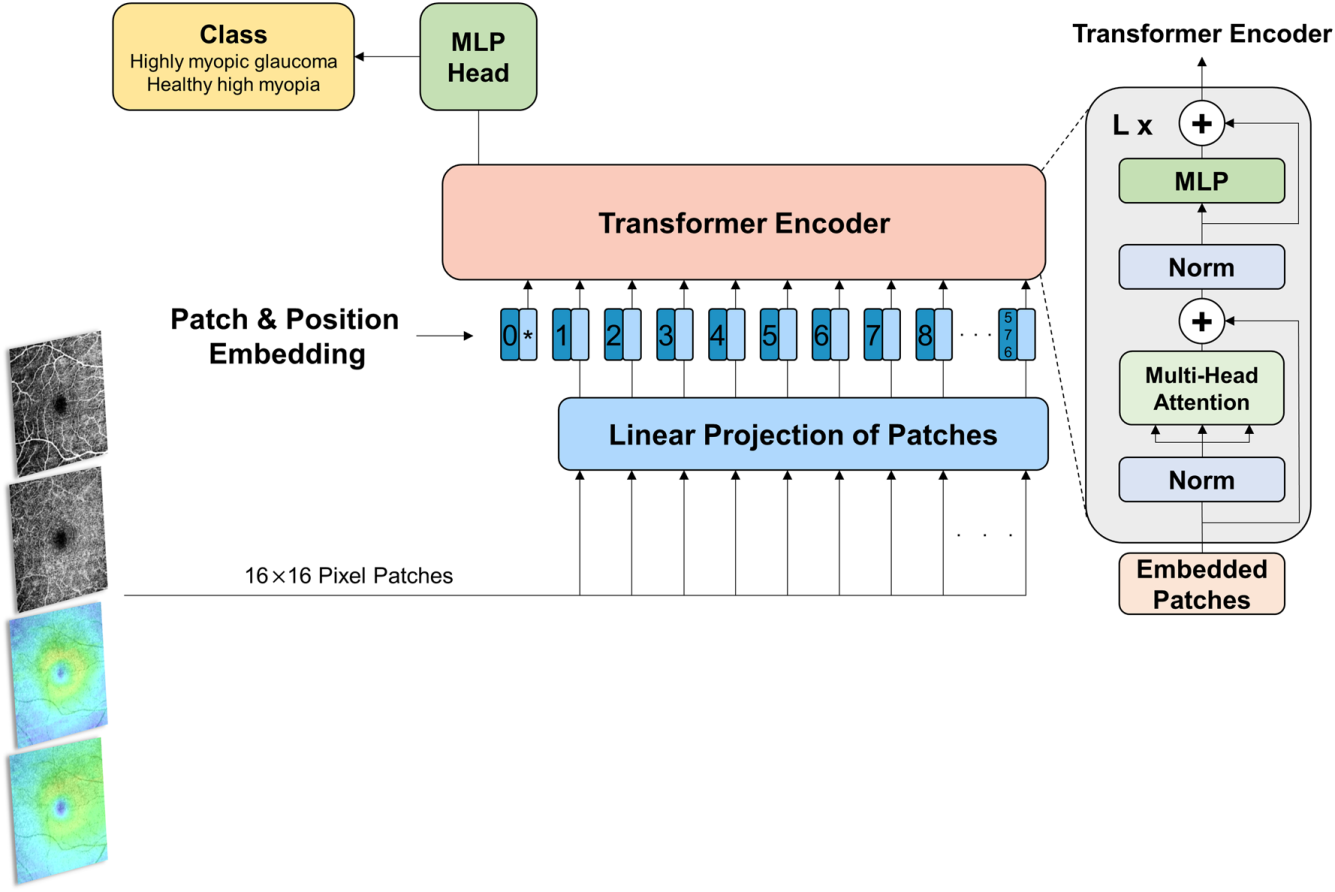
Schematic of Vision Transformer (ViT) model. MLP, multi-layer perceptron.

A schematic of the ViT model is shown in Fig. 3. Images of 384 × 384 pixels were fed into the network (which compensates for image signal intensity variation with internal preprocessing) in the form of multiple patches of 16 × 16 pixels (a total of 576 patches). They were then flattened to 1-dimensional vectors as inputs to the Transformer encoder. Through linear projections, a total of 768 (16 × 16 × 3) 1-dimensional vectors were converted to 768-dimension embedding vectors (i.e., patch embeddings). The other vectors, classification tokens, were employed as 1-dimensional representation vectors for the images. Positional information was retained by adding position embeddings to the patch embeddings. The encoder consists of alternating layers of multi-head self-attention and multi-layer perceptron (MLP) blocks, wherein LayerNorm (LN) and residual connections are applied before and after every block, respectively^20^. In multi-head self-attention, 768 vectors were multiplied by the weight matrices ${W}_{q}, {W}_{k},{W}_{v}$ to obtain, respectively, query (Q), key (K), and value (V). A dot product value of Q and K was normalized by the softmax function, and a final attention value was obtained by multiplying the softmax of ${Q}^{T}\cdot$ K by V. The output of the encoder was passed through the MLP head, and thereby, the score for each class was obtained.

#### Hardware specifications

CPU: Intel(R) Xeon(R) Gold 5120 CPU @ 2.20 GHz.

GPU: Tesla V100 32 GB ×2.

#### Software specifications

Preprocessing: OpenCV 3.4.2

DL libraries: Pytorch 1.7.1, Python 3.7

### Visual explanation of deep learning model’s decisions

To visualize the regions of the images that had contributed to the trained DL model’s decisions, we generated heatmaps using Grad-CAM^61^, which was extracted from the first LN of the last block of the Transformer encoder. In detail, Grad-CAM uses the gradient information flowing into the last layer to assign importance values to each neuron for a particular decision of interest, thereby producing a heatmap highlighting the important regions in the image^61^.

### External validation

To validate the DL model’s performance, we used external datasets from Seoul National University Bundang Hospital, an independent and remote external institute. A total of 108 pairs of macular OCTA and OCT images from 108 eyes (92 eyes with highly myopic glaucoma and 16 eyes with healthy high myopia) were used to differentiate highly myopic glaucoma from healthy high myopia.

### Statistical analyses and performance evaluation

Descriptive statistics were obtained to represent the data, and the Student's t-test and Chi-square test were used to compare the results between the subgroups. To evaluate the performance of the DL model, the AUC, accuracy, sensitivity, and specificity with 95% CI were calculated respectively for the OCTA and OCT image datasets. The sensitivity and specificity of the ROC curves were obtained by thresholding the output value of the designed network after normalizing (0–1) by the softmax function. DeLong’s test was used to compare AUCs between any two parameters^62^. In interpreting the DL model’s performance, we referred to the results from previous studies^33,56,60^ that had investigated the diagnostic ability of SS-OCT in glaucoma; Wan et al.^33^ reported that the mean and regional inner macular thicknesses measured with SS-OCT performed well for discrimination between glaucomatous and healthy eyes, with AUCs ranging from 0.81 to 0.93. Also, Yang et al.^60^ reported that SS-OCT wide-angle and peripapillary RNFL thickness measurements performed well for detecting glaucomatous damage, with AUCs of 0.88 and 0.89, respectively. Our group’s former study^56^ also showed that the wide-field RNFL thickness map using SS-OCT performed well in distinguishing eyes with preperimetric glaucoma and early perimetric glaucoma from healthy eyes, the RNFL thicknesses of the 7 clock-hour, inferior and inferotemporal macular ganglion cell analyses showing the largest AUCs, which ranged from 0.809 to 0.865. The data was analyzed using the Statistical Package for the Social Sciences version 25.0 (IBM Corp., Armonk, NY, USA), R statistical package version 3.3.3 (R Foundation for Statistical Computing, Vienna, Austria), and Python version 3.7 (Python Software Foundation, Wilmington, DE, USA). In the analyses, *P* values < 0.05 were considered statistically significant.

## Supplementary Information

Supplementary Information.

## Data Availability

The data that support the findings of this study are available from the corresponding author upon reasonable request.
