# Supplementary material for: Diagnostic ability of macular microvasculature with swept-source OCT angiography for highly myopic glaucoma using deep learning
Source: Sci Rep. 2023 Mar 30;13:5209. doi: 10.1038/s41598-023-32164-9 (PMC10063664; doi:10.1038/s41598-023-32164-9)

# Supplementary Information

## Diagnostic ability of macular microvasculature with swept–source OCT angiography for highly myopic glaucoma using deep learning

Yun Jeong Lee,[1] Sukkyu Sun,[2] Young Kook Kim,[1] Jin Wook Jeoung,[1] Ki Ho Park[1]

[1]Department of Ophthalmology, Seoul National University Hospital, Seoul National University College of Medicine, Seoul, Korea

[2]Biomedical Research Institute, Seoul National University Hospital, Seoul, Korea

**Supplementary Table S1.** Comparison of demographics and clinical characteristics between highly myopic glaucomatous eyes and healthy highly myopic eyes with external datasets

| | **Highly Myopic Glaucoma (n = 92)** | **Healthy High Myopia (n = 16)** | ***P* Value** |
|---|---|---|---|
| Age (yrs) | 42.5 ± 8.7 | 41.6 ± 7.0 | 0.832* |
| Female, no. (%) | 35 (38.0) | 5 (31.3) | 0.604† |
| Intraocular pressure (mmHg) | 12.7 ± 2.4 | 13.1 ± 2.7 | 0.586‡ |
| Spherical equivalent (D) | -8.2 ± 2.0 | -7.9 ± 2.1 | 0.675‡ |
| Axial length (mm) | 27.0 ± 0.7 | 26.7 ± 0.6 | 0.135* |
| Central corneal thickness (μm) | 541.5 ± 26.6 | 549.5 ± 17.6 | 0.336‡ |
| Visual field mean deviation (dB) | -5.07 ± 4.91 | -0.30 ± 1.18 | **<0.001*** |

Data are mean ± standard deviation unless otherwise indicated.
Boldface indicates *P* < 0.05.
*Mann-Whitney U test.
†Chi-square test.
‡Student's t–test.

**Supplementary Table S2.** Performance of deep learning model in distinguishing highly myopic glaucoma from healthy high myopia with external datasets

| | **AUC (95% CI)** | **Accuracy (95% CI)** | **Sensitivity (95% CI)** | **Specificity (95% CI)** |
|---|---|---|---|---|
| OCTA SCP | 0.873 (0.807–0.940) | 0.807 (0.724–0.873) | 0.462 (0.154–0.539) | 0.903 (0.365–0.903) |
| OCTA DCP | 0.794 (0.687–0.902) | 0.815 (0.734–0.880) | 0.308 (0.154–0.615) | 0.957 (0.591–0.957) |
| OCT GCL+ | 0.970 (0.941–1.000) | 0.935 (0.871–0.974) | 0.923 (0.423–1.000) | 0.939 (0.854–0.988) |
| OCT GCL++ | 0.843 (0.722–0.964) | 0.817 (0.696–0.905) | 0.353 (0.294–0.824) | 1.000 (0.163–1.000) |

*AUC* area under the receiver operating characteristic curve, *CI* confidence interval, *DCP* deep capillary plexus, *GCL+* ganglion cell layer + inner plexiform layer, *GCL++* retinal nerve fiber layer + ganglion cell layer + inner plexiform layer, *OCT* optical coherence tomography, *OCTA* optical coherence tomography angiography, *SCP* superficial capillary plexus.

**Supplementary Table S3.** Comparison of OCT angiography devices

| **Device (Manufacturer)** | **OCT Modality** | **OCTA Algorithm** | **Scanning Speed** | **Light Source** | **Axial/Transverse Resolution** |
|---|---|---|---|---|---|
| AngioVue RTVue XR Avanti (Optovue) | Spectral-domain | SSADA | 70,000 A-scans/sec | 840 nm | 5/15 µm |
| Spectralis OCT Angiography (Heidelberg Engineering) | Spectral-domain | FSADA | 85,000 A-scans/sec | 870 nm | 7/14 µm |
| PLEX Elite 9000 (Carl Zeiss Meditech) | Swept-source | OMAG | 100,000 A-scans/sec | 1,040–1,060 nm | 6.3/20 µm |
| DRI OCT Triton (Topcon) | Swept-source | OCTARA | 100,000 A-scans/sec | 1,050 nm | 8/20 µm |

*DRI* deep range imaging, *FSADA* full-spectrum amplitude-decorrelation angiography, *OCT* optical coherence tomography, *OCTA* optical coherence tomography angiography, *OCTARA* optical coherence tomography angiography ratio analysis, *OMAG* optical microangiography, *SSADA* split-spectrum amplitude-decorrelation angiography.

**Supplementary Table S4.** Comparison of demographics and clinical characteristics between internal and external datasets

| | Highly Myopic Glaucoma | | *P* Value | Healthy High Myopia | | *P* Value |
|---|---|---|---|---|---|---|
| | **Internal Dataset (n = 203)** | **External Dataset (n = 92)** | | **Internal Dataset (n = 57)** | **External Dataset (n = 16)** | |
| Age (yrs) | 46.6 ± 9.8 | 42.5 ± 8.7 | **0.001*** | 42.7 ± 14.1 | 41.6 ± 7.0 | 0.658* |
| Female, no. (%) | 81 (39.9) | 35 (38.0) | 0.803† | 28 (49.1) | 5 (31.3) | 0.204† |
| Spherical equivalent (D) | -8.1 ± 1.3 | -8.2 ± 2.0 | 0.082* | -8.5 ± 1.6 | -7.9 ± 2.1 | 0.099* |
| Axial length (mm) | 26.9 ± 0.7 | 27.0 ± 0.7 | 0.223* | 26.8 ± 0.6 | 26.7 ± 0.6 | 0.705‡ |
| Visual field mean deviation (dB) | -5.38 ± 5.49 | -5.07 ± 4.91 | 0.648* | -0.02 ± 1.67 | -0.30 ± 1.18 | 0.619* |
| | **Internal Dataset** | | | **External Dataset** | | |
| Glaucomatous eyes, no. (%) | 203 (78.1) | | | 92 (85.2) | | 0.119† |

Data are mean ± standard deviation unless otherwise indicated.

Boldface indicates *P* < 0.05.

*Student's t–test.

†Chi-square test.

‡Mann-Whitney U test.

**Supplementary Figure S1.** Distribution plots comparing demographics and clinical characteristics between internal and external datasets for highly myopic glaucomatous eyes (**A,C,E,G,I**) and healthy highly myopic eyes (**B,D,F,H,J**). (**A,B**) Age. (**C,D**) Gender. (**E,F**) Spherical equivalent. (**G,H**) Axial length. (**I,J**) Visual field mean deviation. (**K**) Diagnosis (glaucoma, normal)

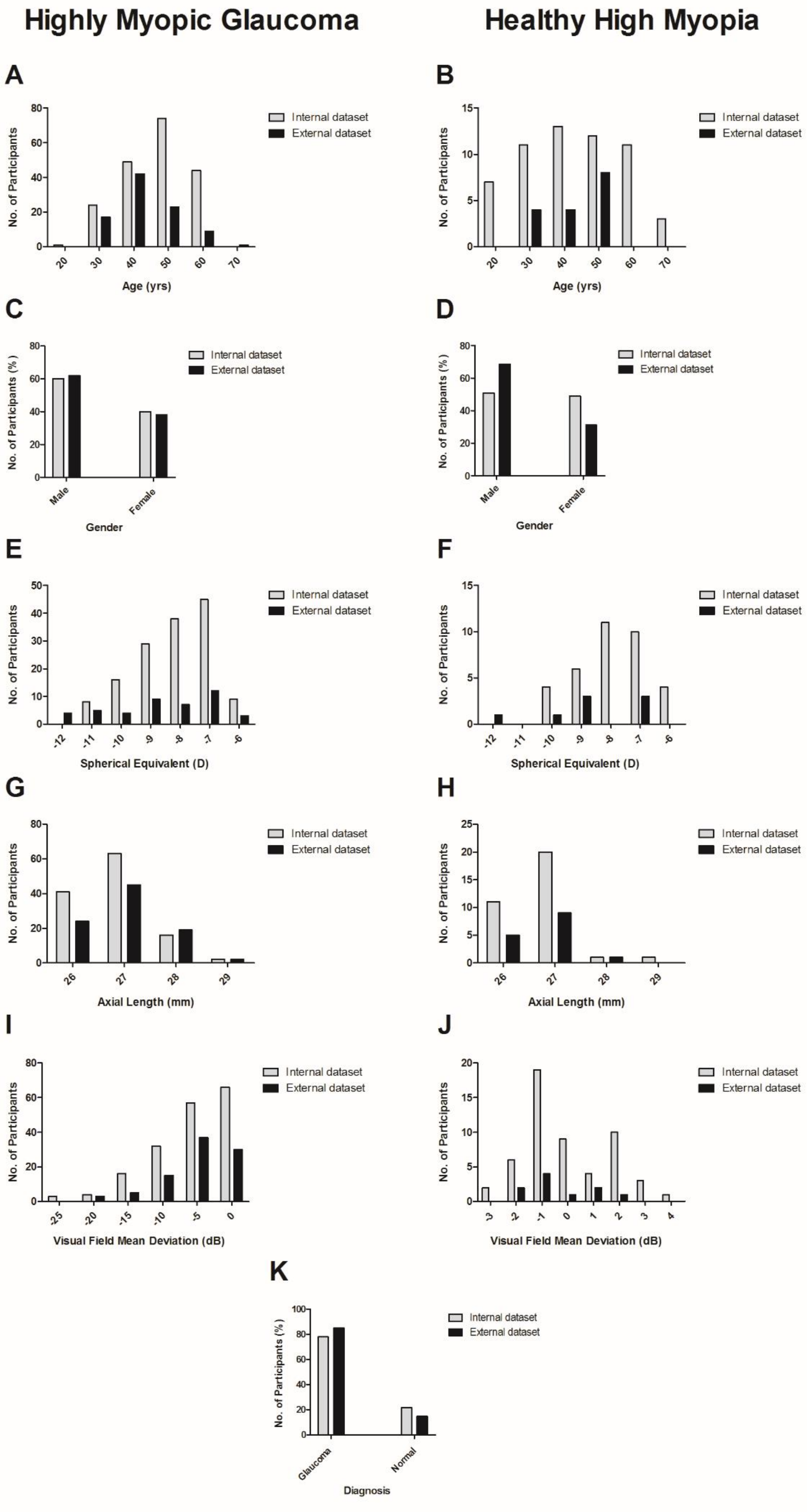

Supplement: Supplementary file 1 — Supplementary Information. [file 41598_2023_32164_MOESM1_ESM.pdf]
